# Effect of live oud music on physiological and psychological parameters in patients undergoing cardiac surgery

**DOI:** 10.21542/gcsp.2019.17

**Published:** 2019-09-20

**Authors:** Merna Luis, Ramy Doss, Basel Zayed, Magdi Yacoub

**Affiliations:** 1 Aswan Heart Centre, Aswan, Egypt; 2 Music and Art therapist, Ellenhorn, Massachusetts, USA

## Abstract

**Background.** Music therapy has emerged as a promising evidence-based adjuvant method of intervention. This study aims to assess the effect of live oud music on physiological and psychological parameters in patients undergoing cardiac surgery, pre- and post-operatively.

**Methods.** Twelve patients undergoing cardiac operations were randomly allocated into either intervention group or control group, six patients in each group. Patients in the intervention group listened to 20 minutes of improvised and personally customized live oud music before and after surgery while patients in the control group heard the normal hospital sounds. While anxiety scores were assessed preoperatively, vital signs and pain scores were assessed postoperatively together with serum levels of cortisol, which was used as a surrogate marker of the stress response.

**Results.** In the intervention group, pain scores and respiratory rates showed statistically significant reduction after listening to music (P values of 0.043 and 0.034 respectively). Additionally, heart rates, anxiety scores and serum cortisol levels showed borderline significant reduction in patients who listened to music with P values, 0.063, 0.066 and 0.068 respectively. These changes were not found in the control group.

**Conclusions.** This preliminary study suggests a role of live oud music therapy in decreasing stress response of the patients undergoing cardiac surgery, in addition to its positive effects on pain perception and anxiety scores.

## Introduction

Over the past few decades, music therapy has emerged as promising evidence-based adjuvant treatment addressing various organic and psychiatric illnesses^1–5^.

Major surgical procedures are known to be associated with considerable pain, anxiety and hemodynamic disturbances. Thus, the use of music therapy as an adjuvant therapy to alleviate perioperative anxiety and alter pain perception has been of a particular interest.

In this study, we aimed to explore the effect of live music intervention on various hemodynamic parameters, anxiety and pain perception scores as well as serum cortisol level as a surrogate markers of stress response in patients undergoing cardiac surgery.

While various studies have discussed the efficacy of passive music therapy using recorded music as an adjuvant therapeutic intervention^6^, much less is known about the efficacy of live music interventions in patients undergoing surgery.

To the best of our knowledge, this study represents the first randomized trial to examine effects of music therapy on Egyptian patients, using Arabic and Middle Eastern music.

## Methods

The study took place at Aswan Heart Centre, Aswan, Egypt, between November 2018 and February 2019, using a single-centre, prospective, open- label randomized controlled trial design. According to the inclusion criteria, twelve adult patients were eligible to participate in the study. They were 18 years old or above, undergoing either elective septal myectomy, valve replacement, or coronary artery bypass graft operations. Patients were excluded if they were unconscious, sedated, haemodynamically unstable or on inotropic support. They were also excluded in case of co-morbidities that might interfere with the study results, such as advanced kidney or liver diseases.

After consenting, the patients were randomly allocated into either experimental or control group. Patients in the experimental group received sessions of 20–30 minutes of live relaxing oud music performed by a trained oud player. Melodies, duration, volume, tempos and scales were selected based on the individual explicit preference of every patient in addition to the oud player’s insight. Structure of the music therapy sessions was planned ahead with a credentialed music therapist.

The first session was performed two hours before surgery. Anxiety scores were assessed using a certified Arabic translation of Hamilton Anxiety assessment scale^7^ before and after the music intervention for the experimental group. Postoperatively, after patients were extubated and weaned of vasopressors, music sessions were resumed on the 1st and 2nd post-operative days. Systolic and diastolic blood pressure, heart rate, respiratory rate, oxygen saturation and pain scores (using the Visual Analogue Scale^8^) were recorded before and after music 24 and 48 hours postoperatively. Additionally, serum cortisol levels were measured using electrochemiluminescence immunoassay (ECLIA) within 30 minutes before and after music on the 1st day.

For the control group, music therapy sessions were replaced with periods of rest, in which the patients heard the typical hospital sounds. Same measurements were recorded. Data were collected by personnel blinded to group assignments.

Statistical analysis was performed using SPSS version 18.0 (SPSS Inc., Chicago, Illinois). Due to the non-normal distribution and small sample size, data were expressed as median and interquartile range. Repeated measures were compared using Wilcoxon Signed Ranks Test. P values less than 0.05 were considered statistically significant.

## Ethical considerations

The study was approved by the local ethical committee of Aswan Heart Centre. All participants gave informed written consents after being explicitly provided with information about the study.

## Results

Twelve patients were included in this preliminary analysis, six men and six women of median age 48 years. Six patients were in the experimental group and six in the control group. They were comparable regarding their age, sex and type of surgery.

**Table 1:** Results for experimental and control groups.

	Pre- intervention^*^	Post- intervention	*P* value
***HR (bpm) 1st day***			
experimental	85 (73.50–89.00)	82 (72.50–86.50)	0.063
control	96 (92.00–102.00)	95 (90.00–104.50)	1
***HR (bpm) 2nd day***			
experimental	80 (67.50–90.00)	79 (66.50–79.00)	0.059
control	92 (83.50–102.00)	95 (86.00–98.50)	0.892
***RR (bpm) 1st day***			
experimental	30 (26.50–32.00)	26 (21.00–28.50)	0.043
control	26 (25.00–38.50)	32 (24.50–33.50)	0.893
***Anxiety (0–56)***			
experimental	14 (10.25–21.25)	7 (6.00–7.25)	0.066
control	8 (2.75–12.00)	8 (2.75–12.00)	1
***Pain (0–10) 1st day***			
*experimental*	7 (6.00–7.50)	5 (3.50–5.50)	0.034
*control*	7 (3.00–9.00)	7 (3.00–9.00)	1
***Pain (0–10) 2nd day***			
experimental	5 (3.00–6.00)	3 (1.00–4.50)	0.039
control	5 (1.50–7.50)	5 (1.50–7.50)	1
***S- Cortisol (nmol/L)***			
experimental	1170 (399.50–1552.00)	945 (287.50–1524.00)	0.068
control	592 (243.20–1410.00)	880 (392.40–1376.00)	0.515

**Notes.**

* Values expressed as median (inter-quartile range IQR).

*P <0.05 is considered statistically significant.

*HR, heart rate, RR, respiratory rate.

In the intervention group, statistically significant reduction in respiratory rates was observed in the first postoperative day (P <0.043) as well as pain scores in the first (P <0.034) and second days (P <0.039). Additionally, borderline significant reductions in heart rates in first (P <0.063) and second (P <0.059) days, anxiety scores (P <0.066) and serum cortisol levels (P <0.068) (Table 1). In the control group, no statistically significant differences were found in any measured parameter. In both groups, no significant differences were demonstrated regarding systolic or diastolic blood pressure or oxygen saturation levels.

## Discussion

**Figure 1.**
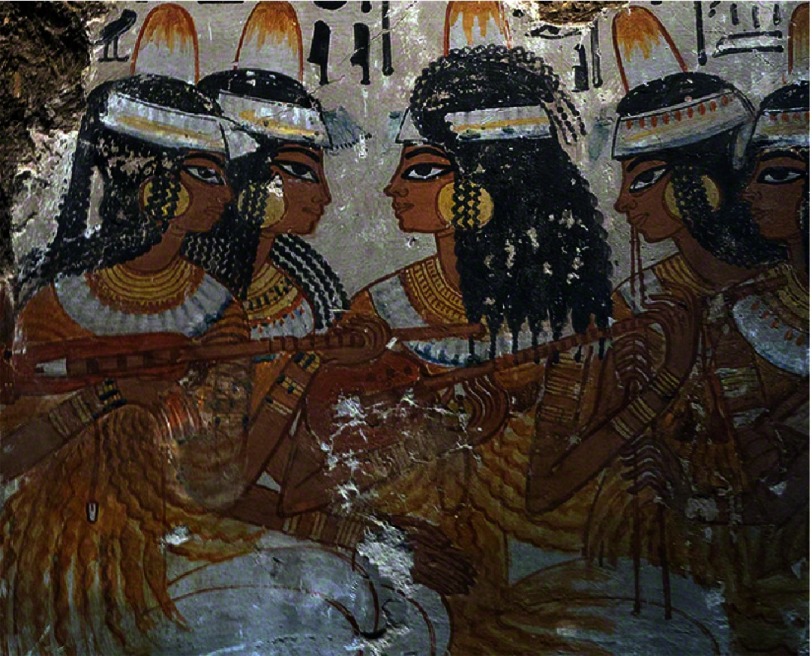
Egyptian lute or oud players. Fresco found in Thebes, from the tomb of Nebamun, a nobleman in the 18th Dynasty of Ancient Egypt (c. 1350 BC).

In Ancient Egypt, harp was used for healing purposes to address many illnesses of mind and body^9, 10^ (Figure 1). It is mentioned that Imhotep, one of the greatest physicians in Ancient Egypt^11, 12^ used music therapy and constructed a sanatorium for this purpose^10^. Use of music therapy, especially for pain relief, was also evident hundreds of years ago during the Islamic Era^13^.

Reseach in this field has been growing over the past few decades, with a view of integrating music therapy into the conventional medical practice.

The results of this preliminary study emphasize some positive effects of live music therapy on various physiological and psychological parameters. Clinically, music resulted in statistically significant reduction in heart rate and respiratory rate. This is consistent with the findings of previous studies which presented evidence of decreased heart and respiratory rates^14–19^.

However, in contrast to these studies, no statistically significant changes in blood pressure were found in our study. With regard to the psychological parameters, music therapy significantly decreased preoperative anxiety and postoperative pain scores. These effects were also proved in many previous studies^14, 15, 20–30^.

In some systematic reviews including all randomized clinical trials that compared music intervention with standard care to standard care only, researchers concluded that music intervention might have a beneficial effect on preoperative anxiety^14, 15, 20–24^, especially when patients are involved in the music selection process^15^.

**Figure 2.**
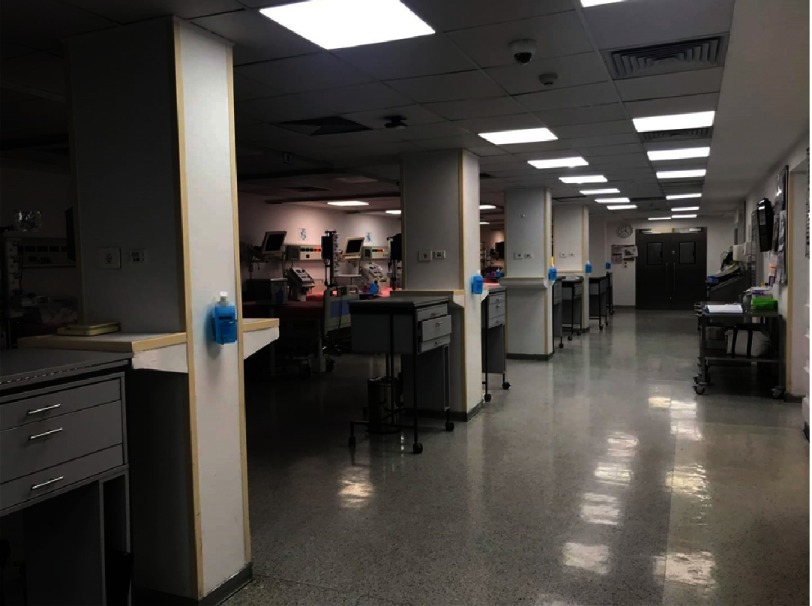
ICU unit at Aswan Heart Centre.

Many other RCTs have been done to assess the effect of music intervention on different kinds of pain in children and adults, most of them concluded that music can be used as a beneficial adjuvant modality for reducing pain perception^25–30^ (Figure 2).

As cortisol is a known component of the endocrine stress response to surgeries^31^, serum cortisol level was used in this study as a surrogate biochemical marker to stress response and was found to decline significantly in response to live oud music. Similarly, Leardi et al found a statistically significant reduction of serum cortisol level in patients who had undergone day surgeries, after listening to music^32^. Regarding stress response to cardiac surgery, a statistically significant difference of serum cortisol levels between the music group and control group was found^33^.

While the exact mechanism of the positive central effects of music remains unknown, some studies have attributed this to positive distraction^34^, cognitive role of pain control^35^, and increased levels of beta-endorphins^36^ resulting in positive emotional responses and mood changes^37–40^.

Functional neuroimaging studies have allowed better understanding of the unique response of brain to music. Studies have highlighted modulation of metabolic activity of brain structures involved in emotional regulation in response to music, giving rise to autonomic and endocrine responses^39, 41–43^. Increased dopamine activity in the mesolimbic system occurs in response to anticipation and listening to the preferable kind of music associated with peak emotional response^44^.

**Figure 3.**
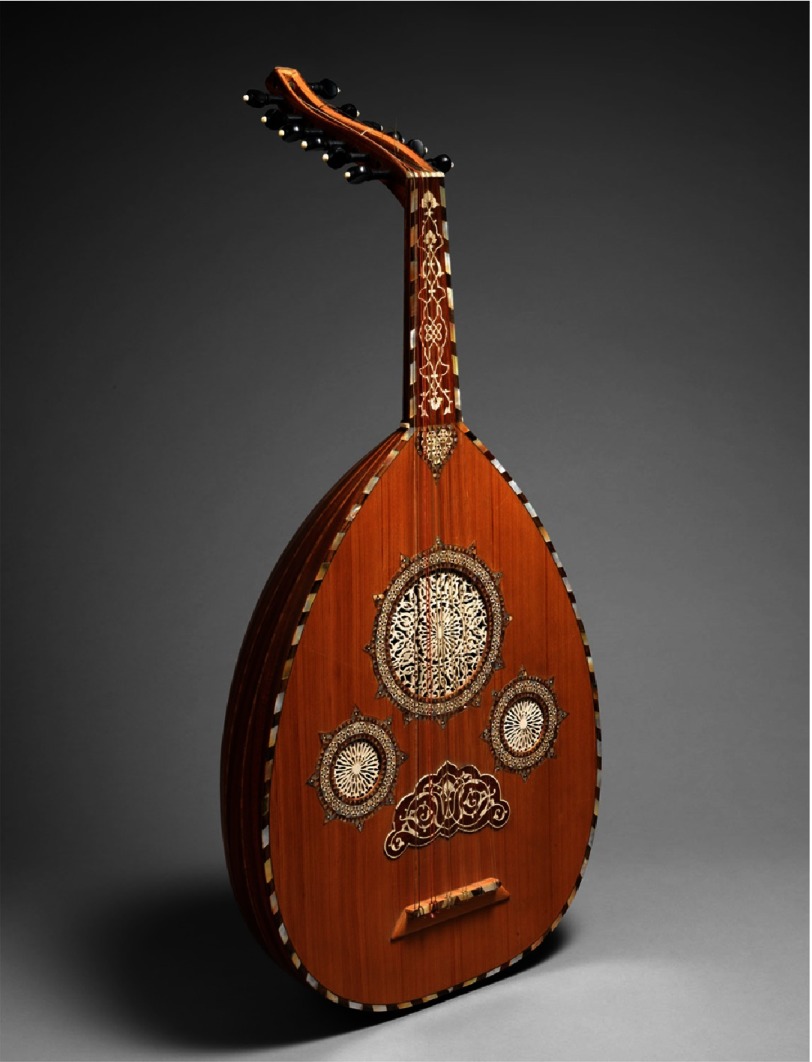
A typically traditional Egyptian oud.

In this context, we selected a lute-type musical instrument deeply rooted in Arabian culture, the ‘oud’, given its soothing effect and familiarity to Arab and Egyptian patients (Figure 3). Carl Jung talked about the concept of collective subconscious and how society makes use of the inherited creative material for meaning making and soothing. The collective unconscious is “the part of the psyche that retains and transmits the common psychological inheritance of mankind. These symbols are so ancient and unfamiliar to modern man that he cannot directly understand or assimilate them”^45^. Perhaps this is the reason why it is soothing to use an authentic instrument. Moreover, live music involves having the therapist with the patient which brings empathy together with the concept of “here and now” to the relationship^46^.

**Figure 4.**
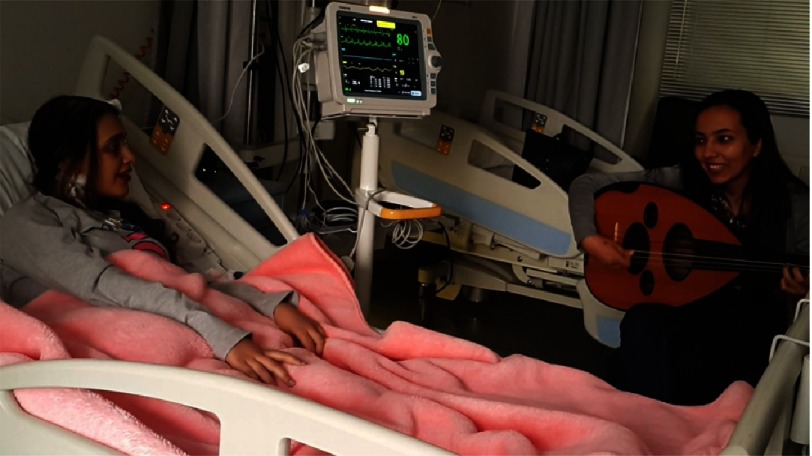
A live oud therapy session taking place at the Aswan Heart Centre.

Furthermore, we categorized the music therapy sessions into two major sections; firstly, patients’ favorite songs were played upon their request, and secondly, improvisation using a unique, culturally- based approach called the ‘Maqam’ system. Maqam is a scale system in the Arabic and Middle Eastern musical heritage. The wide range of tones produced in Maqam is significantly important to underscore. The specific healing texture brought from the variety of tones produces an abundance of scale colors which allows the composer to improvise to express a deeper and more emotionally and socially connected sound (Figure 4).

We felt it was necessary to develop a therapeutic alliance which is used to give patients a sense of choice and help them feel responsible and safe. This is in line with what Carl Rogers states about the therapist-patient relationship: “The relationship is one which provides the client with the opportunity of making responsible choices”^46^.

This method enabled us to achieve an individualized approach facilitating the active involvement and engagement of patients in the music making process. During each session, patients chose between four different scales, performed by the musician, without being informed of the name, purpose or nature of each scale. Then improvisation within the chosen maqam was performed which might helped the patients express specific subjective emotions.

Interestingly, it was observed that the most commonly chosen scales were Bayati and Kurd while Hijaz was the second popular choice. Moreover, the popular songs requested by the patients lied within the same scales. This observation might reflect the nature of the music genre preferred to Arab and Egyptian native listeners which might help reach a peak response and a maximum benefit.^44^

## Study limitations

The study is limited by the small number of patients which might cause some false negative results, lack of follow up for sustained clinical benefits and lack of clinical translation of the neuro-hormonal changes. Finally, problems related to questionnaire-based studies, such as response bias, were faced.

Permission for the reproduction of the patient’s identifying features in Figure 4 was freely given and obtained by the authors.

## Conclusion

The results of this preliminary study suggest a role for live oud music therapy in decreasing stress response of the patients undergoing cardiac surgery, showing statistical significant reduction in respiratory rate, heart rate, pain and anxiety scores as well as cortisol levels. It might also emphasize the importance of improvised, patient- centered live music intervention. However, further studies are needed to compare efficacy of live versus passive music interventions, and to provide a complete picture of Arab and Egyptian patients’ therapeutic musical preferences.
